# Development of a high-precision bladder hyperthermic intracavitary chemotherapy device for bladder cancer and pharmacokinetic study

**DOI:** 10.1186/s12894-019-0543-8

**Published:** 2019-12-03

**Authors:** Mingchen Ba, Shuzhong Cui, Hui Long, Yuanfeng Gong, Yinbing Wu, Kunpeng Lin, Yinuo Tu, Bahuo Zhang, Wanbo Wu

**Affiliations:** 1 Intracelom Hyperthermic Perfusion Therapy Center, Cancer Hospital of Guangzhou Medical University, No. 78 Hengzhigang Road, Guangzhou, Guangdong 510095 People’s Republic of China; 2 Department of Pharmacy, Guangzhou Dermatology Institute, Guangzhou, Guangdong People’s Republic of China

**Keywords:** Hyperthermic intracavitary chemotherapy, Bladder cancer, Survival, Animal model

## Abstract

**Background:**

Bladder hyperthermic intracavitary chemotherapy (HIVEC) has good effectiveness for bladder cancer, but conventional HIVEC systems lack precision and convenient application. To test the safety of a new HIVEC device (BR-TRG-II-type) in pigs and to perform a preliminary clinical trial in patients with bladder cancer.

**Methods:**

This device was tested on six pigs to optimize the temperature and time parameters. Then, 165 patients (HIVEC after transurethral resection (TUR), *n* = 128; or HIVEC, *n* = 37) treated between December 2006 and December 2016 were recruited. Mitomycin C (MMC) was the chemotherapeutic agent. A serum pharmacokinetic study was performed. The primary endpoints were tumor recurrence, disease-free survival (DFS), and cumulative incidence rate (CIR) during follow-up. The adverse effects were graded.

**Results:**

The animal experiment showed that 45 °C for 1 h was optimal. HIVEC was successful, with the infusion tube temperature stably controlled at about 45 °C, and outlet tube temperature of about 43 °C in all patients, for three sessions. Serum MMC levels gradually increased during HIVEC and decreased thereafter. The mean DFS was 39 ± 3.21 months (ranging from 8 to 78 months), and the DFS rate was 89.1% during follow-up. No adverse events occurred.

**Conclusion:**

The use of the BR-TRG-II-type HIVEC device is feasible for the treatment of bladder cancer. Future clinical trials in patients with different stages of bladder cancer will further confirm the clinical usefulness of this device.

**Trial registration:**

chictr.org.cn: ChiCTR1900022099 (registered on Mar. 252,019). Retrospectively registered.

## Background

Bladder cancer ranks among the top five malignant tumors worldwide, with over 70,000 new patients diagnosed with bladder cancer each year in the United States [1]. The standard procedure for bladder cancer removal is still transurethral resection (TUR) or surgical resection [2], but recurrence is always a major concern. As much as 80% of patients with bladder cancer confined to bladder epithelium will experience disease recurrence, and up to 45% of patients with invasion of lamina propria and 10% with carcinoma in situ will experience disease progression without treatment [3]. Intravesical Bacillus Calmette-Guerin (BCG) is recommended as adjuvant therapy [4], but recurrence and progression occurs in a substantial proportion of patients [5]. Mitomycin C (MMC) is also recommended as adjuvant treatment, but its efficacy is limited [6–9]. TUR or surgical resection alone cannot be performed microscopically, and systemic chemotherapy has only limited efficacy against bladder cancer [3, 10]. Therefore, preventing recurrence of bladder cancer after TUR and preventing progression in patients unsuitable for TUR or surgical resection remain major problems in oncology [11, 12].

Bladder hyperthermic intracavitary chemotherapy (HIVEC) combines the advantages of local hyperthermia with intracavitary chemotherapy, which have a synergistic or at least additive effect in preventing bladder cancer recurrence post TUR or surgical resection [13–16]. However, available systems have issues in the precision of temperature control to the target site [6–9, 11–14, 16–19], limiting their efficacy and safety [3, 10, 13–18].

The BR-TRG-I-type hyperthermic intraperitoneal perfusion chemotherapy (HIPEC) device is a recent HIPEC device now approved by the Chinese Food & Drug Agency (license number 2009–3260924) and covered by two Chinese patents (ZL2006200613779 and ZL2006200613764). The BR-TRG-I-type HIPEC device has been shown to be safe and effective for the treatment of malignant ascites and peritoneal cancer [20]. The BR-TRG-I-type HIPEC device has been tested for hyperthermic intraperitoneal perfusion chemotherapy [21], but it is not suitable for bladder cancer. On the basis of the BR-TRG-I-type HIPEC device, we developed the BR-TRG-II-type HIVEC device, which has been shown to be safe and efficient in preventing the recurrence of non-muscle invasive bladder cancer (NMIBC) after TUR and prolonging disease-free survival (DFS) [12]. This device allows for a precise temperature (±0.2 °C) and flow (±5%) control [20]. Therefore, the aim of the present exploratory study was to test the safety of the device in pigs, and to perform a preliminary clinical trial in patients with bladder cancer treated.

## Methods

### Animals

Animal experiments with the BR-TRG-II-type HIVEC device were performed using six healthy female experimental pigs (*Sus scrofa domesticus*; 40–50 kg, median 52.6 kg; 4–6 months old, median 5 months) purchased from the Animal Experiments Center of Nanfang Medical University (Guangzhou, China). The experimental animals were sacrificed by intravenous air embolization after receiving general anesthesia with intravenous infusion of propofol (femoral vein, 3–8 ml/h, adjusted according to the condition of the animals). This study was approved by the Ethics Committee of Animal Experiments of Guangzhou Medical University (No. GZMU ECAE 20060326), Guangzhou, China. All animals were handled humanely, and all means were taken to minimize suffering. All experiments were carried out according to the animal experiment principles from the US National Institutes of Health and according to the regulations from the Chinese government.

**Fig. 1:**
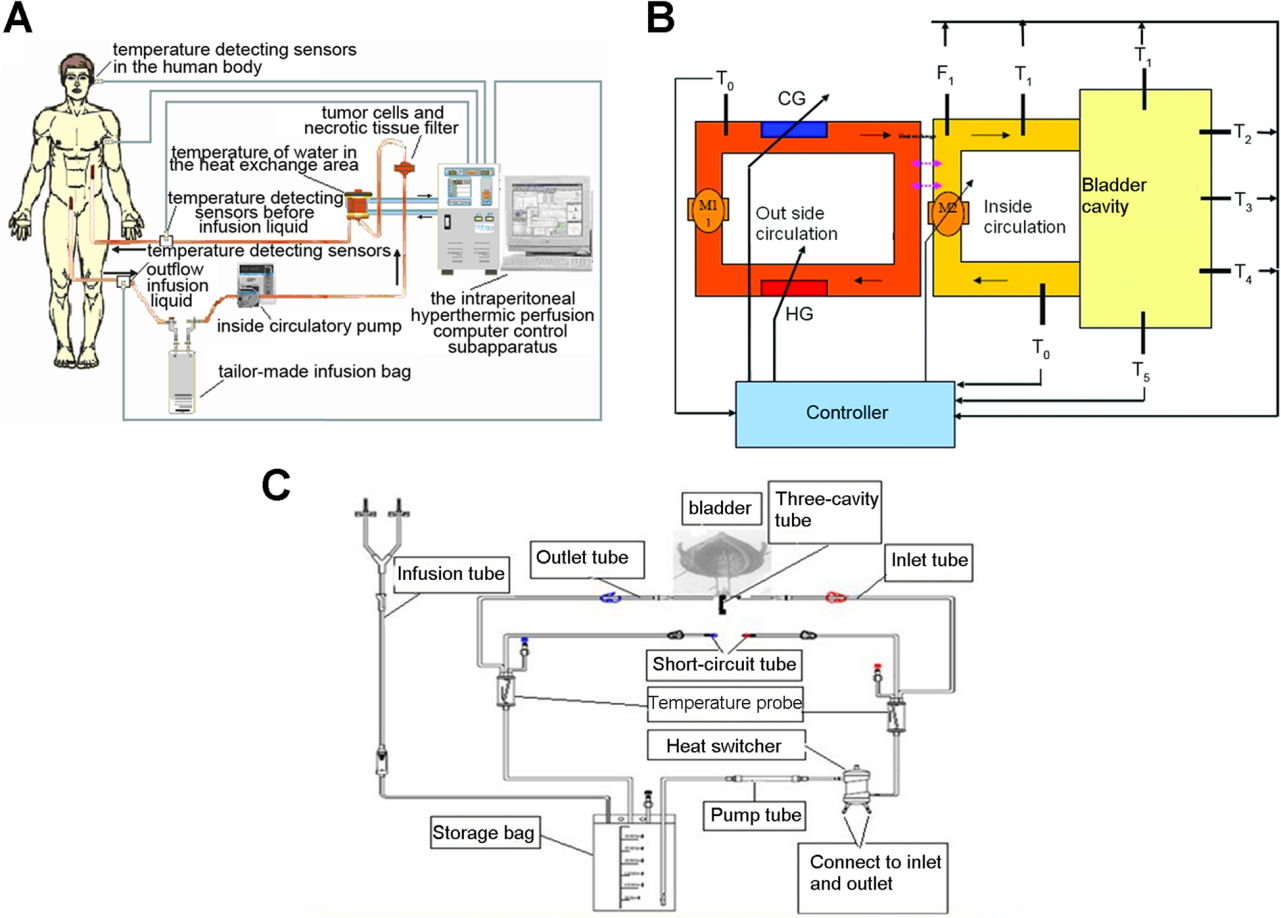
Design of the BR-TRG-II-type HIVEC device. (**a**) Overall use of the device. (**b**) Design of the device. **c**) Schematic diagram of the tubes during HIVEC. HG: heater, maximum power of 4 kW; CG: semi-conductor refrigerator, maximum power of 2 kW; Ti: temperature of water in the heat exchange area of, precision of 0.1 °C; Tii: temperature of the circulatory perfusion liquid before entering the human body, precision of 0.1 °C; Tiii: temperature of the drug solution after exiting from the cavity, precision of 0.1 °C; Fi: flow rate of the circulatory perfusion liquid, precision of 10 mL/min, resolution of 1 mL/min; P: pressure of the drug solution before entering the body, precision of 10 mmHg, resolution of 1 mmHg; T_1_-T_5_: temperature of five parts of the human body; M1: external circulatory pump, constant working rate of 10 L/min; M2: inside circulatory pump, rotating rate, controllable maximum rate of 600 mL/min, precision of 10 mL/min, resolution of 1 mL/min

**Fig. 2:**
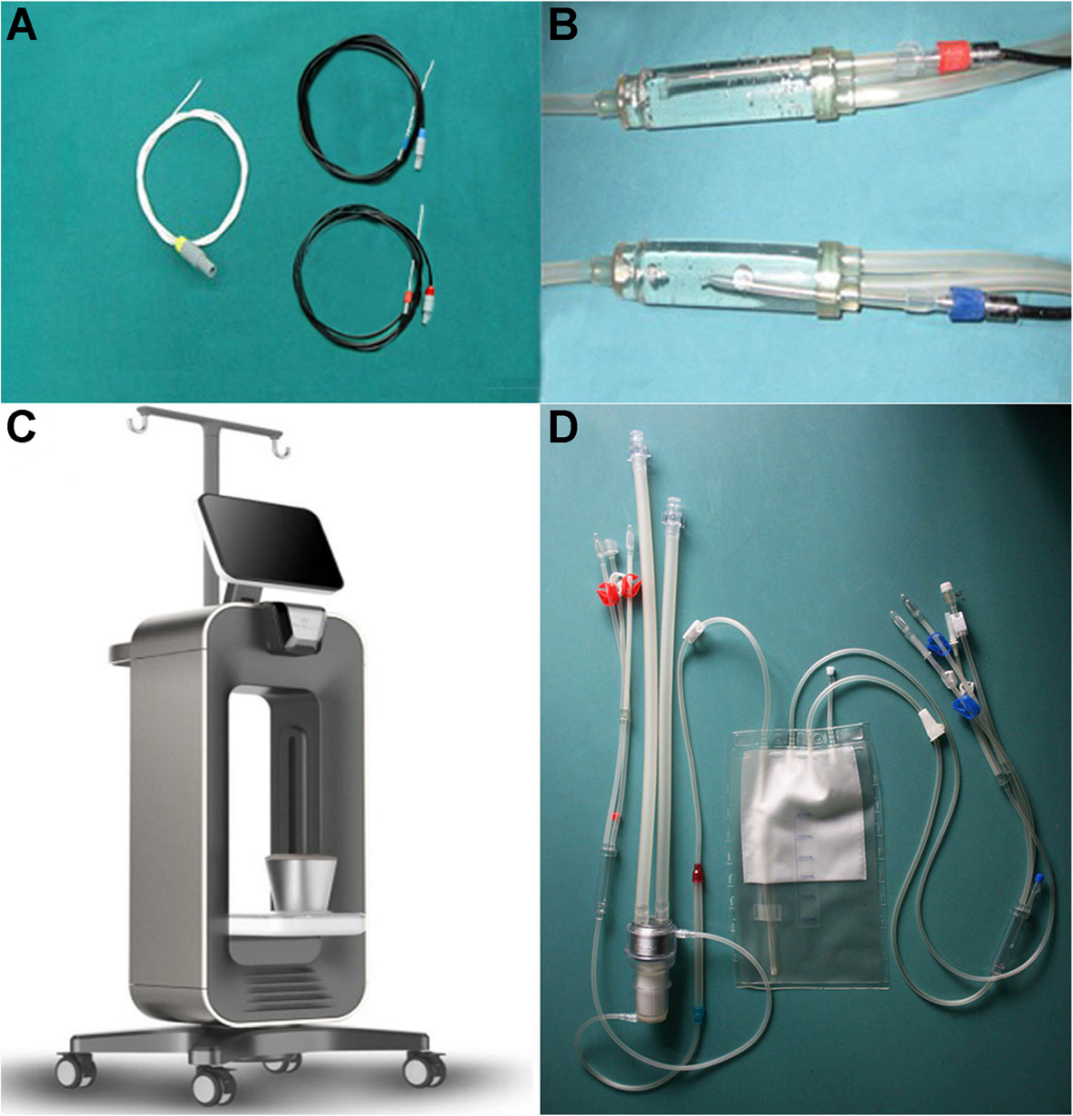
Illustration of the BR-TRG-II-type HIVEC system for HIVEC. (**a**) Temperature-monitoring probes. One tip is placed into a fixed water sac linked to an infusion tube near an inlet (red cap) or outflow catheters (blue cap) at the top of a 24 F 3-way Foley catheter (yellow cap). (**b**) Temperature-monitoring probes location; red cap locates near the infusion catheters, and blue cap locates near the outflow catheters. (**c**) The device. **d**) The tubes

Under endotracheal anesthesia, 24 F 3-way Foley catheters were introduced into the bladder cavity, and 5 ml of warm saline were injected to inflate the sac and fix the catheter in the bladder cavity. The BR-TRG-II-type HIVEC device (Fig. 1) was connected to the catheter and loaded with a solution of 60 mg of MMC (Zhejiang Hisun pharmaceutical Limited by Share Ltd., Hangzhou, China) in 600 ml of sterile saline. The perfusion rate of the MMC solution was set as 150–200 ml/min. The experimental temperature and time (i.e., 44 °C, 46 °C, or 48 °C for 60 min) were set. The treatment temperature during HIVEC was measured by the device using temperature probes inserted in a blind pipe in an inflated water sac linked to an infusion tube near an infusion tube and in a blind pipe in an inflated water sac linked to an outlet tube near the 24 F 3-way Foley catheter (as shown in Fig. 2a b).

Based on the “resource equation” principle [22–24], on the results being observed, and on available resources, six pigs were randomized (random number table prepared by a third-party statistician) to the 44 °C, 46 °C, and 48 °C groups (2 pigs/group). Before HIVEC, the perfusion liquid was adjusted to the proper temperature. Any temperature change was monitored closely by the temperature probes. HIVEC was performed once a week for 3 weeks. The Foley catheter was pulled out after every session. The bladder mucosa changes after HIVEC were assessed by cystoscopic observation under endotracheal anesthesia [13, 19]. The observer was blind to grouping.

### Clinical trial

Patients with bladder cancer were prospectively recruited from December 2006 to December 2016 at the Intracelom Hyperthermic Perfusion Therapy Center of the Cancer Hospital of Guangzhou Medical University. This study was retrospectively registered (chictr.org.cn: ChiCTR1900022099). The patients received TUR + HIVEC or HIVEC according to whether they were suitable or not for TUR [2]; there was no randomization for this part of the study, nor blinding. Bladder cancer was diagnosed and staged by cystoscopic observation, computerized tomography (CT), and/or magnetic resonance imaging (MRI) examination.

The inclusion criteria were: 1) ≥18 years of age; 2) diagnosis of bladder cancer by cystoscopic observation, CT, and/or MRI; 3) diagnosis confirmed by histopathological examination of a biopsy specimen; 4) no radiation therapy in the 4 weeks preceding enrollment; and 5) no chemotherapy in the 4 weeks preceding enrollment. The exclusion criteria were: 1) stage Ta bladder cancer; 2) known or possible bladder metastasis from other primary cancer; 3) known or possible bladder tumor expanding through the serosa, invading locally or metastasizing to other organs; 4) known or potential pregnancy; or 5) active inflammation or infection. Based on whether the patients were suitable for TUR or not, TUR + HIVEC or HIVEC was performed. TUR + HIVEC was performed for patients eligible for TUR but not for cystectomy and reconstruction because of comorbidities or incapacity to bear the surgical trauma. In patients unable to bear any surgery, HIVEC was performed.

All treatments were performed by our study team with clinical experience with TUR and HIVEC. This study was approved by the Medical Ethics Committee of the Cancer Hospital of Guangzhou Medical University (No. GZMU ECAE 20060326). Written informed consent was obtained from all patients. Some of the patients included in the present study were also included in a previous study by our group [12], but differences in the selection criteria resulted in different sample sizes and groups of patients between the two studies.

### Transurethral resection

Cystoscopy was performed under epidural anesthesia. A 24-Fr monopolar resectoscope system (Karl Storz, Tuttlingen, Germany) was used for TUR. Under cystoscopy, the profile and margins of the tumor were first defined. Resection was then performed carefully to avoid perforation and wall distention. The emptied bladder was manually manipulated on the pubic symphysis in cases of poorly located tumors. Small and flat lesions were positioned between the resection loop and the end portion of the resectoscope sheath. Hemostasis was carefully performed after tumor removal [13, 19].

### HIVEC

HIVEC was directly performed for patients unsuitable for TUR, or 0–1 days after TUR. To do so, 24 F 3-way Foley catheters were introduced into the bladder cavity for HIVEC, and 5 ml of warm saline were injected to inflate the sac and fix the catheter in the bladder. The BR-TRG-II type high-precision HIVEC device was connected to tubes (Guangzhou Bright Medical Technology Co., Ltd.). A bag containing 60 mg of MMC in 500–700 ml (average 600 ml) of sterile saline, as previously reported [6, 7]. The liquid perfusion rate was set as 150–200 ml/min. The treatment temperature and time were set (i.e., 45 °C for 60 min) according to the patient’s clinical data.

Before HIVEC, perfusion liquid was adjusted to 350–450 ml (average 400 ml) at 45 °C within the bladder according to the perfusion pressure and the patient’s subjective experience. The amount of perfusion fluid within the bladder cavity could be increased or decreased according to the patient’s subjective experience, and the temperature change was monitored closely by the temperature probe. The patient’s vital signs (including blood pressure, heart rate, respiratory rate, and blood oxygen saturation) were monitored using a G3HJ20025 multi-parameter patient monitor (MINDRAY Bio-Medical Electronics Co. Ltd., Shenzhen, China). HIVEC treatment was terminated if any accidents happened, such as treatment temperature > 45 °C or bladder lumen pressure over patients’ tolerance during HIVEC. HIVEC was performed once a week for 3 weeks. The 24 F 3-way Foley catheters were retained for 3–5 days for urine drainage after the first session for observing for eventual bleeding of the TUR wound.

### Pathological examination

In all patients, the tumor tissues were taken before HIVEC or 4 weeks after HIVEC and were observed for histological changes by hematoxylin-eosin (H&E) staining, as described previously [13, 19]. Resected specimens were reviewed by an experienced pathologist blinded to grouping, as described previously [13, 19].

### Pharmacokinetics of MMC

Venous blood (from the venous catheters at 0, 15, 30, 45, 60, 75, and 90 min) and perfusion liquid (from the short circuit outflow catheters of the perfusion system at 0, 15, 30, 45, 60, 75, and 90 min) samples were collected from 12 patients in the two groups. Perfusion liquid, serum, or MMC standard samples (degassed prior to use) (200 μl) were vortexed for 1 min with 200 μl of acetonitrile containing diazepam (20 μg; internal standard). The samples were separated by centrifugation at 15,000 rpm for 15 min. The supernatants (10 μl) were subjected to high-performance liquid chromatography (HPLC, aLC-20AB, Shimazdu, Kyoto, Japan). The stationary phase was Zorbax RP, and C_18_ (250 × 4.6 mm; particle size 5 μm) packed columns. The analysis was performed as previously reported [25], but with some modifications. The mobile phase was a 60:20:20 (%, volume) solution of 50 mM potassium dihydrogen phosphate buffer solution, acetonitrile, and methanol, pH 3.0, and filtered using a 0.22-μm membrane (Millipore, Billerica, MA, USA). Samples were injected at 1.2 ml/min. The absorption wavelength for detection was 210 nm. The column oven temperature was 35 °C. The linear ranges of the standard curves were 0.10–10.0 mg/ml for MMC in the perfusion liquid and 0.50–50.0 ng/ml for MMC in the serum [26, 27].

### Follow-up

Follow-up was performed by urinary cystoscopic observation at 1 month after TUR and then each 3 months for 1 year. These patients underwent abdominal and pelvic CT scans at 3, 6, and 12 months, or when clinically indicated. After 1 year, follow-up was carried out at 6-month intervals or less frequently if the patients remained without evidence of disease.

### Endpoints

The primary endpoints were tumor recurrence (diagnosed by cystoscopic observation, CT, or MRI), DFS, and cumulative incidence rate (CIR) during follow-up. The adverse effects of the anticancer drugs were graded according to the Common Toxicity Criteria of the National Cancer Institute for Adverse Events (CTCAE) [28].

### Statistical analysis

In the animal part, a minimum of six animals was deemed necessary to reach any conclusion. For the human part, the sample size was not calculated because there was no randomization. This is a convenience sampling of all patients who met the criteria during the study period and agreed to participate in the study. All continuous data were tested for normal distribution using the Kolmogorov-Smirnov test, are presented as mean ± standard deviation, and were analyzed using the Student’s t-test (intergroup comparisons) or repeated measure ANOVA with the LSD post hoc test (intragroup comparisons). Categorical data are presented as frequencies and were analyzed using the Fisher’s exact test. CIR and DFS were analyzed using the Kaplan-Meier curve method with the log-rank test. Data were analyzed using SPSS 19.0 (IBM, Armonk, NY, USA). Two-sided *P*-values < 0.05 were considered statistically significant.

## Results

### Animal experimental data

The animal experiment showed that when using the device set at 44 °C for 1 h, a temperature of about 43 °C was achieved in the intravesical cavity without affecting the vital signs of the animals. The bladder mucosa showed slight pathological changes and returned to normal by 1 h after HIVEC. Setting HIVEC at 46 °C for 1 h achieved an intravesical temperature of about 45 °C and caused slightly increased blood pressure and heart rate, along with bladder mucosa hyperemia and edema, which returned to normal by 3 days after the final HIVEC. Setting the HIVEC at 48 °C for 1 h achieved an intravesical temperature of about 47 °C and caused significant increases in blood pressure and heart rate, along with bladder mucosa pathological changes that did not return to normal by 1 week after HIVEC. Therefore, 45 °C for 1 h was used in the clinical study.

### Characteristics of the patients

One hundred and sixty-five patients with bladder cancer were eventually enrolled in this study. There were 108 males and 57 females, with a median age of 51 years (ranging from 37 to 76 years). Of these patients, 128 cases underwent HIVEC after TUR (including four patients with recurrent bladder cancers with a disease-free period of 3–6 months post-TUR), while 37 received HIVEC. There were no significant differences in age, gender, disease course, and tumor location, stage, and size between the two groups (all *P* > 0.05) (Table 1).

**Table 1:** Characteristics of the patients with bladder cancer

	HIVEC+TUR (n = 128)	HIVEC (n = 37)	P
Age (years)	50.7 ± 1.9 (37–66)	51.6 ± 2.3 (39–66)	0.07
Sex, *n* (%)			
Male	86 (67.2)	22 (59.5)	0.09
Female	42 (32.8)	15 (40.5)	0.07
Disease course (days)	11.4 ± 1.3	11.3 ± 1.6	0.07
Tumor location, *n* (%)			
Side wall	35 (27.3)	1 (2.7)	0.06
Posterior wall	27 (21.1)	2 (5.4)	0.08
Top area	19 (14.8)	1 (2.7)	0.08
Triangle area	10 (7.8)	17 (45.9)	0.06
≥ two tumors	37 (28.9)	16 (43.2)	0.07
Tumor size, *n* (%)			
≥ 0.5 cm	66 (51.6)	28 (75.6)	0.07
< 0.5 cm	62 (48.4)	9 (24.4)	0.09
Tumor stage, *n* (%)			
Tis	13 (10.2)	0	0.06
T_1_	73 (57.0)	9 (24.3)	0.06
T_2_	42 (32.8)	28 (75.7)	0.08
Tumor differentiation, n (%)			
G_1_	41 (32.0)	11 (29.7)	0.08
G_2_	54 (42.2)	14 (37.8)	0.08
G_3_	33 (25.8)	12 (32.4)	0.75

HIVEC: bladder intracavitary hyperthermic perfusion chemotherapy

### Gross outcomes of HIVEC

**Fig. 3:**
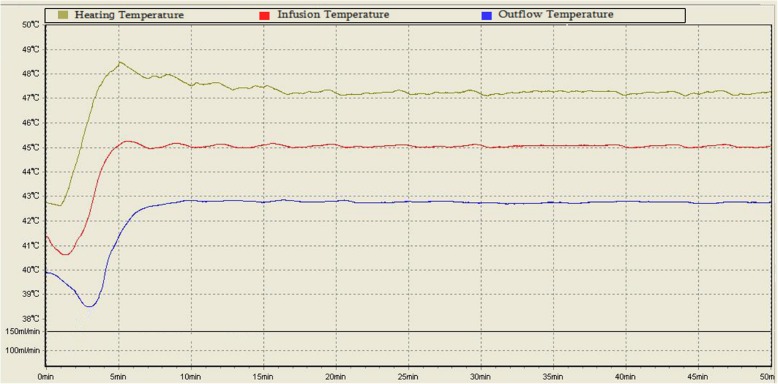
Temperature curves of the infusion fluid and outflow fluid during HIVEC

HIVEC was successful, with the infusion tube temperature stably controlled at about 45 °C, and an outlet tube temperature of about 43 °C (Fig. 3). All patients tolerated three sessions of HIVEC. For all patients in the HIVEC groups, gross hematuria stopped after 2 days after the first HIVEC, but slight hematuria lasted for up to one week following the first treatment.

### Pharmacokinetics of MMC

**Fig. 4:**
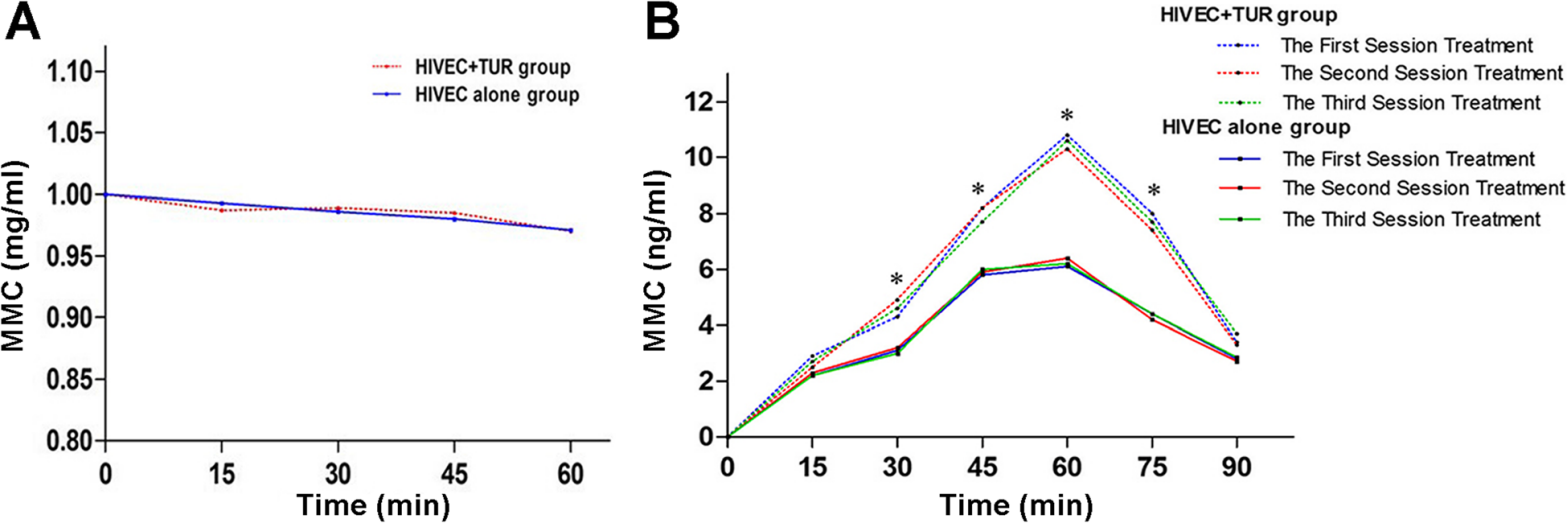
Mitomycin C (MMC) levels in the perfusion fluid and serum in the HIVEC+TUR and HIVEC groups. (**a**) Dynamics of MMC concentration in the perfusion fluid. (**b**) Dynamics of MMC concentration in serum. *P < 0.05

The MMC concentration in the bladder perfusion fluid gradually decreased during treatment from 1 mg/ml to 0.967 mg/ml in the HIVEC + TUR group and 0.970 mg/ml in the HIVEC groups (P > 0.05) (Fig. 4a). The MMC concentration in the serum gradually increased during HIVEC treatment in both groups, to 4.32 ± 0.11, 7.86 ± 0.14, 10.08 ± 0.21, and 7.56 ± 0.16 ng/m at, 15, 30, 45, 60, and 75 min in the HIVEC + TUR group, which were significantly higher than in the HIVEC group (3.01 ± 0.09, 5.78 ± 0.11, 5.98 ± 0.12, and 5.66 ± 0.13 ng/ml, respectively) (Fig. 4b). The MMC concentration in serum decreased after HIVEC, being all below 3.28 ± 0.08 ng/ml at 90 min (Fig. 4b).

### Cystoscopic and histological observation

**Fig. 5:**
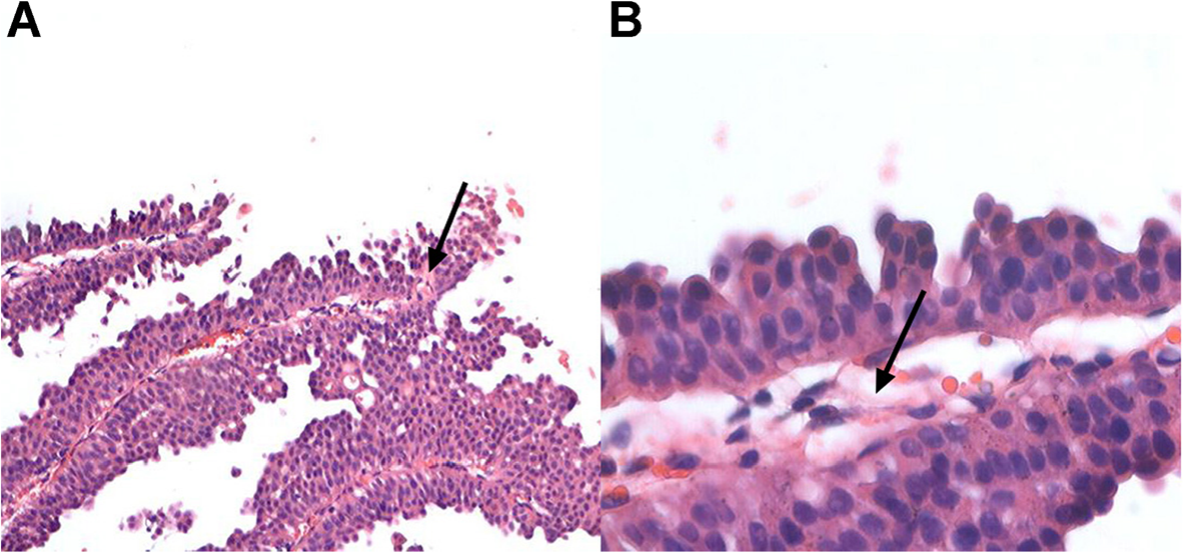
Histological change after HIVEC in the HIVEC groups. (**a**) Histological examination showed cancer cell degenerative necrosis, inflammatory cells (sometimes including numerous eosinophils (arrow)) (hematoxylin and eosin, × 100). (**b**) Histological examination showed tumor-infiltrating the lamina propria, but with complete necrosis accompanied by local vascular changes (such as necrosis and thrombosis (arrow) in the small tumor vessels, and hemorrhage into the stroma (hematoxylin and eosin staining, × 400)

In the HIVEC + TUR group, cystoscopy showed no viable tumor lesions, except in 12 patients who had T2 diseases; the lesions were showing as grey-white slough on the bladder mucosa around the lesions, accompanied by congestion and edema. In the HIVEC group, all patients showed cystoscopic findings consistent with the findings observed in those with residual lesions in the HIVEC + TUR group. Pathological examination showed that the lesions presented degenerative necrosis and inflammatory cells such as eosinophils infiltrating the lamina propria (Fig. 5a) Complete necrosis accompanied by local vascular changes (such as necrosis and thrombosis) in the tumor small vessels were also observed, as well as stromal hemorrhage (Fig. 5b).

### Follow-up

**Fig. 6:**
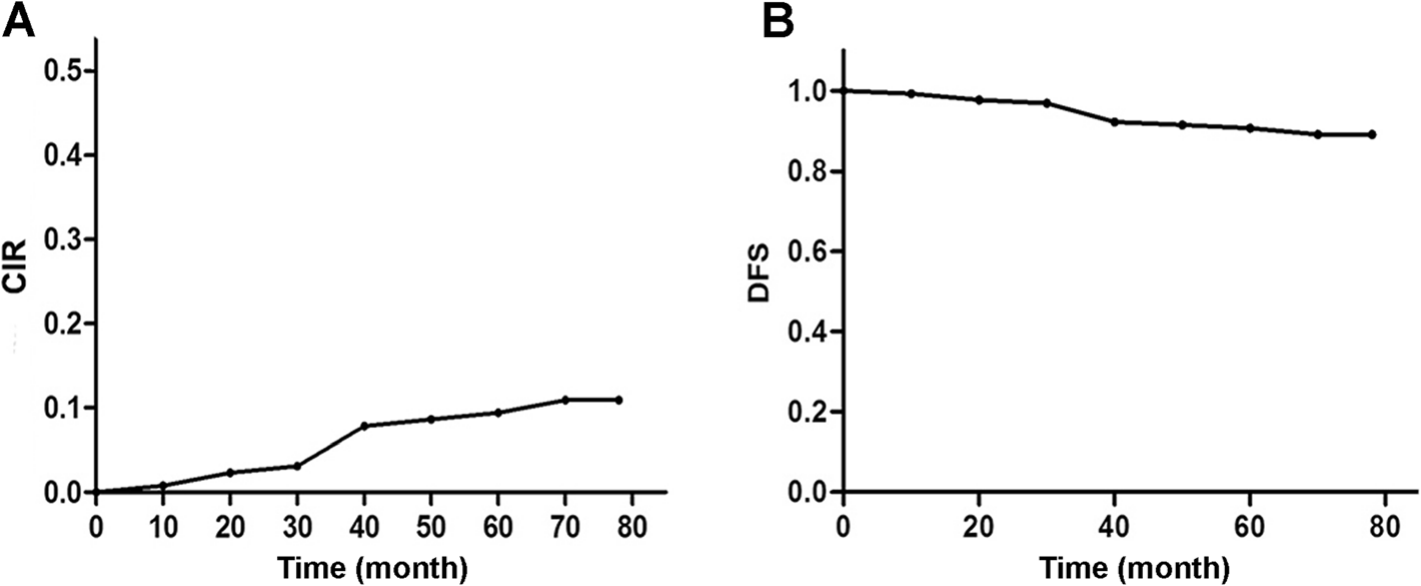
Kaplan-Meier curves of cumulative incidence rate (CIR) and disease-free survival (DFS) for patients with bladder HIVEC after TUR. **a** Kaplan-Meier curves of cumulative incidence rate (CIR), and the CIR was 10.9% (14 out of 128 patients) for patients with bladder HIVEC after TUR. **b** Kaplan-Meier curves of disease-free survival (DFS), and the mean DFS was 39.0±1.2 months (ranging from 8 to 78 months), and the DFS rate was 89.1% during follow-up for patients with bladder HIVEC after TUR

All patients were followed for at least 6 months. The median follow-up was 41.9 months (6.5 to 110 months) for the HIVEC + TUR group and 42.3 months (10.5 to 99.7 months) for the HIVEC group (*P* > 0.05). All patients were still alive at the moment of writing this paper. In the HIVEC + TUR group, cystoscopic observation showed tumor recurrence in 14 patients after HIVEC, which included nine patients with remaining tumor after HIVEC + TUR. The CIR was 10.9% (14 out of 128 patients). The mean DFS was 39 ± 3.21 months (ranging from 8 to 78 months), and the DFS rate was 89.1% during follow-up (Fig. 6a b). In the HIVEC group, bladder tumor numbers were decreased in 78.4% (29/37) patients or disappeared in 16.2% (6/37) patients; 51.35% (19/37) patients could undergo TUR 1–2 months after HIVEC.

### Adverse effect

No gastrointestinal events or bone marrow suppression occurred. Laboratory tests showed no significant changes in blood, electrolytes, and liver and kidney functions after treatment in all patients. There were no genitourinary or dermatologic adverse reactions such as bladder spasms, chemical cystitis, or chemical irritation of scrotal skin in all patients.

## Discussion

Bladder HIVEC has good effectiveness for bladder cancer [7–13, 19, 29], but the precision and convenient application of conventional HIVEC systems are unsatisfactory. Therefore, this study aimed first to examine the safety of a new HIVEC device (BR-TRG-II-type) in pigs, and then to perform a preliminary clinical trial in patients with bladder cancer. The results showed that the BR-TRG-II-type HIVEC device could be used for the treatment of bladder cancer. Nevertheless, the results need to be confirmed in patients with different cancer stages. Of note, HIVEC is an experimental treatment that is not currently included in any treatment guideline. Nevertheless, a number of studies using different, less accurate HIVEC systems have been performed [7–13, 19, 29], and this approach could eventually be included in bladder cancer treatment guidelines. Since cystectomy cannot be performed in some patients [30], TUR combined with intravesical HIVEC could be a good option for these cases.

The most important physicians’ concern during any treatment involving hyperthermia is setting an adequate temperature. Indeed, too high temperature will cause thermal damage, while too low temperature will not achieve the optimal therapeutic effects. Complicating the issue is the fact that in HIVEC, different body compartments will require different temperatures. In HIPEC, the intra-abdominal temperature generally does not exceed 43 °C because of the risk of intestinal adhesions and obstruction. On the other hand, for intravesical HIVEC, the temperature is usually set to 45 °C because bladder mucosa damage recovers fast [3, 9, 12, 14, 26, 31]. In the present paper, the preclinical experiments in pigs showed that the BR-TRG-II-type HIVEC device could meet the requirements for precise temperature control, heating, and cooling, hence ensuring stable, secure, reliable, and convenient application in the clinical setting. Furthermore, the experiments in pigs revealed that intravesical HIVEC at 45 °C for 1 h did not affect vital signs; the bladder mucosa showed only slight pathological changes, which returned to normal within 1 h after HIVEC completion.

Following the animal study, the preliminary clinical study and the results showed that HIVEC using the BR-TRG-II-type HIVEC device was feasible in a clinical setting, without any cases of postoperative deaths or serious complications. Gross hematuria disappeared after HIVEC in all patients, bladder lesions were smaller or even disappeared, and > 50% of the patients who were initially unsuitable for TUR got the chance to receive TUR after HIVEC, as supported by previous reports that revealed the advantages of HIVEC [4–9, 11–19, 32].

The use of intravesical MMC was tried in a number of previous studies [6–9, 11, 12]. Those studies used an MMC dose of at most 60 mg in 60 ml of saline (i.e., MMC at 1 mg/ml), which is considered safe and effective in clinical practice [6–9, 11, 12], but the knowledge of the vesical absorption rate of chemotherapeutic drugs is limited at best. In fact, high MMC concentrations has little relationship with the therapeutic effects [11, 15, 16, 18, 26]. In addition, the biological effects of cytotoxic drugs under high temperatures are poorly known. In the absence of concrete knowledge, we tested the use of continuous circulation of a fixed dose of chemotherapeutic drugs (60 mg) in a volume of 500–700 ml. In this preliminary trial, this dose was safe and effective. The absorption of MMC is related to damage to the bladder mucosa during intravesical HIVEC, and MMC concentration remains excessively high in the absence of mucosal damage, potentially leading to cystitis [6]. Taking those considerations into account, MMC concentration was 0.1 mg/ml in the present study, which is much higher than the 0.002 mg/ml (10 mg/5000 ml) used in HIPEC and systemic chemotherapy. In the preliminary studies, using different concentrations of MMC did not improve the therapeutic effects, but there were no adverse events either. Nevertheless, in the present study, intravesical HIVEC using high MMC concentration ensured a high, constant, and sustained local chemotherapeutic drug concentration thought to achieve the best chemotherapeutic efficacy. In the present study, the MMC concentration in the perfusion fluid was gradually decreased over HIVEC time, probably due to urine dilution or/and systemic absorption. Indeed, serum MMC levels increased during HIVEC and decreased after HIVEC, but previous studies in humans indicated that serum MMC concentrations after HIVEC do not reach a critical toxic threshold [8, 9]. In the present study, serum MMC concentrations peaked at 60 min, and the serum MMC levels stayed below the toxic value, and the half-life of MMC is 30–50 min [7, 8]. Accordingly with the higher absorption in the presence of mucosal damage, serum MMC concentrations in the TUR+ HIVEC group were significantly higher than those in the HIVEC group.

The effects of temperature on mucosal blood vessels could lead to plasma exudation and interstitial hemorrhage. There is also a risk that these vascular changes exacerbated the direct thermal injury to the lamina propria, sometimes resulting in necrosis with exfoliation of the epithelium [6, 7, 14, 18]. In the HIVEC group, complete necrosis accompanied by local vascular changes (such as necrosis and thrombosis) in the small tumor vessels and hemorrhage into the stroma was indeed observed. Because tumor vessels are more susceptible to thermal injury than normal tissue vessels, these changes may be responsible, at least in part, for inhibiting bladder cancer growth [7, 8].

It is recommended that HIVEC is performed for at least seven sessions, but there is no standard for intravesical HIVEC in China and worldwide. In the present study, only three HIVEC sessions were performed, mainly to study process and to prevent delays of further treatments. Additional treatments were performed for non-responsive patients after three sessions, which could include surgical resection, arterial embolization thermotherapy, intravesical chemotherapy plus immunotherapy, or intravesical HIVEC plus immunotherapy. Additional trials are necessary to determine the best treatment strategies for the treatment of bladder cancer and the exact place of intravesical HIVEC in those strategies.

This device is not without limitation. Since it is based on conductive heating, the temperature of the fluid can be accurately maintained, but the exact temperature of the bladder mucosa cannot be guaranteed due to heat loss by diffusion in adjacent tissues and blood circulation. Meanwhile, this study only tested the feasibility and safety of this device, as well as MMC pharmacokinetics. Because the incidence of bladder cancer is relatively low in China, the inclusion criteria of this study were relatively strict, leading to a relatively small sample size spanning over many years. Future studies with more patients will be performed to validate these results. A control group will also be included.

## Conclusion

In conclusion, it is feasible to use the BR-TRG-II-type HIVEC device for patients with bladder cancers. This treatment tool has good prospects for widespread clinical application in patients with bladder cancer.

## Data Availability

The datasets used and/or analyzed during the current study are available from the corresponding author on reasonable request.

## Abbreviations

BCG: Bacillus Calmette-Guerin
CIR: Cumulative incidence rate
CT: Computerized tomography
DFS: Disease-free survival
HIVEC: Hyperthermic intracavitary chemotherapy
MMC: Mitomycin C
MRI: Magnetic resonance imaging
TUR: Transurethral resection
